# Timing of cisplatin administration for chemoradiotherapy in transgenic mice bearing lens tumors

**DOI:** 10.3892/or.2014.3202

**Published:** 2014-05-20

**Authors:** SHOJI KAKU, NORICHIKA USHIODA, HIROSHI ISHII, TAKASHI MURAKAMI, KENTARO TAKAHASHI, YUICHIRO NAKAI, KOICHIRO SHIMOYA, TAKAFUMI NAKAMURA

**Affiliations:** 1 Department of Obstetrics and Gynecology, Kawasaki Medical School, Okayama, Japan; 2 Department of Obstetrics and Gynecology, Shiga University of Medical Science, Shiga, Japan

**Keywords:** cisplatin, irradiation, SV40 T antigen, transgenic mice

## Abstract

Cisplatin-based concurrent chemoradiotherapy (CCRT) has become a standard treatment for cancer of the uterine cervix. However, there have been no investigations into the optimum timing for administration of anticancer drugs using animal models. The aim of the present study was to determine the appropriate timing for administration of the anticancer drug cisplatin in relation to delivery of radiation by assessing the antitumor activity and adverse effects of 3 different regimens in αT3 transgenic mice bearing lens epithelial tumors. CCRT showed the strongest antitumor activity. There was a significant difference between CCRT and administration of cisplatin before radiotherapy (neoadjuvant therapy) with regard to the apoptotic effect detected by TUNEL staining, but there was no significant difference between CCRT and administration of cisplatin after radiotherapy (adjuvant therapy). Assessment of adverse effects showed that there were no significant differences in the mortality rate, weight loss, anemia and leukopenia among the 3 regimens. In conclusion, these findings obtained in an animal model suggest that cisplatin should probably not be administered before irradiation, since the antitumor effect is significantly weaker than that of CCRT or administration after irradiation, while adverse effects are similar.

## Introduction

Human papillomavirus (HPV) contributes to the development of cancer of the uterine cervix. It has been reported that the E6 oncoproteins of high-risk HPV types inhibit the activity of p53 tumor suppressor protein, while the E7 oncoproteins of high-risk HPV types inhibit pRB tumor suppressor protein (1,2). Cervical cancer is the second most frequent cancer among women worldwide (3) and concurrent chemoradiotherapy with cisplatin has become standard treatment for locally advanced cervical cancer (4–7). It has been confirmed that cisplatin-based concurrent chemoradiotherapy (CCRT) significantly decreases the risk of mortality due to cervical cancer by 30–50% (8–11), and also improves both disease-free survival and overall survival. However, virtually all chemotherapy agents enhance radiation damage to normal tissues, leading to severe adverse effects of concurrent chemoradiotherapy (12,13).

There has been a lack of animal studies on the timing of administration of anticancer agents. Although *in vitro* studies using cell lines can evaluate efficacy (14–16), adverse effects cannot be properly evaluated. The aim of the present study was to determine the most appropriate timing for the administration of cisplatin with radiation through comparison of the neoadjuvant, concurrent chemoradiotherapy (CCRT) and adjuvant strategies by evaluating efficacy and adverse effects in αT3 transgenic mice (αT3 mice) with undifferentiated lens epithelial tumors induced by the T antigen of SV40, which is a DNA virus resembling HPV types 16 and 18 (HPV16/18) that cause cervical cancer (17,18).

## Materials and methods

### Animals

**Figure 1:**
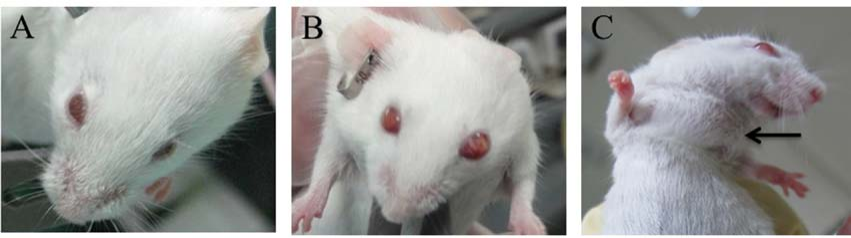
Lens tumors in αT3 mice. (A) The tumor was confined inside the eyeball at 8 weeks of age. (B) The tumor showed extraocular infiltration at 16 weeks of age. (C) The tumor destroyed the eyeball and metastasized to the cervical lymph nodes (black arrow) at 52 weeks of age.

We produced αT3 mice that developed crystalline lens epithelial tumors (17,18). The mechanism of transformation by SV40 T antigen (TAg) of is similar to that by the E6/E7 oncoproteins of HPV16/18 since both TAg and these oncoproteins inhibit the activity of the p53 and pRB tumor suppressor proteins (19). These mice developed lens dysplasia at the embryonic stage and carcinoma *in situ* was observed at 8 weeks after birth. The tumors subsequently showed intraocular invasion (at 16 weeks of age), extraocular invasion (at 32 weeks of age), and metastasis to lymph nodes and other organs (after 52 weeks of age) (Fig. 1).

All procedures were performed in accordance with the Guide for the Care and Use of Laboratory Animals and were approved by the Committee on Animal Experimentation of Kawasaki Medical School. All mice had access to standard rodent chow (NMF; Oriental Yeast Co., Ltd., Japan) and water *ad libitum*, and were housed under pathogen-free conditions in a temperature-controlled animal room with a 12-h light/dark cycle.

### Harvesting of specimens

The mice (N=88; body weight, 28.5±6.5 g) were sacrificed at 32–36 weeks of age as extraocular invasion occurred after 32 weeks. Animals were anesthetized by intraperitoneal (i.p.) injection of sodium pentobarbital (40 mg/kg), blood was collected from the internal jugular vein and the mice were euthanized. Then the eyeballs were carefully resected, fixed in 4% formalin, embedded in paraffin and cut into 4 μm sections. These sections were deparaffinized and stained with hematoxylin and eosin (H&E) staining or were processed for terminal deoxynucleotidyl transferase dUTP nick end-labeling (TUNEL) staining.

### Chemotherapy

Cisplatin (Nippon Kayaku, Tokyo, Japan) was reconstituted with sterile 0.9% saline in a laminar air-flow hood under sterile conditions. Our preliminary experiment showed that the 50% lethal dose (LD_50_) of cisplatin was 16 mg/kg, therefore animals received i.p. chemotherapy with cisplatin at a dose of 2 mg/kg (1/8 of the LD_50_). This dose was approximately equivalent to the clinical dose used for treatment of cervical cancer in humans (40 mg/m^2^) (20) based on the ratio of mass and body surface area between mice and adult human females (21).

### Irradiation

Whole-body irradiation was performed using an MBR-1520R3 X-Ray generator (Hitachi Medical Co., Tokyo, Japan) and the mice received daily fractions of 2.0 Gy from day 0 to 4 (total, 10.0 Gy). The radiation dose and schedule were selected to be similar to those used to treat cervical cancer in humans. (In our preliminary experiment, mice received 5.0–10.0 Gy of whole-body irradiation as a single dose and animals administered 10.0 Gy died within two weeks.)

### Treatment plan

To determine the optimum timing for administration of cisplatin and irradiation, we divided the mice into an irradiation-first group (adjuvant group), a concurrent chemoradiotherapy group (CCRT group) and a cisplatin-first group (neoadjuvant group). Three control groups were also studied, and they received no treatment, cisplatin alone and irradiation alone. Specimens were obtained at three weeks after administration of cisplatin or after starting irradiation (three weeks after starting the second treatment in the neoadjuvant and adjuvant groups).

The mice were divided into the following six groups. Group 1 (N=11) was the untreated control group, Group 2 (N=17) was the cisplatin control group that received i.p. cisplatin on day 0, and Group 3 (N=18) was the irradiation control group that received 2 Gy/day from day 0 to 4. Group 4 (N=14) was the CCRT group, which received i.p. cisplatin on day 0 and radiation at 2 Gy/day on days 0–4. Group 5 (N=13) was designated as the irradiation-first group, and received radiation at 2 Gy/day on days 0–4 and was administered i.p. cisplatin on day 7. Group 6 (N=15) was designated as the cisplatin-first group, and received i.p. cisplatin on day 0 and radiation at 2 Gy/day on days 7 to 11. In all groups, specimens were harvested on day 20. Before treatment (on day 0) and after treatment (on the day of harvesting), the body weight and eyeball size in all mice were measured.

To investigate the antitumor activity of each treatment, we determined the reduction rate of eyeball diameter and assessed apoptosis of tumor cells by TUNEL staining. To investigate adverse effects, we assessed the mortality rate, the changes of body weight, and the hemoglobin and leukocyte count. The hemoglobin was measured in venous blood obtained at the time of sacrifice using an ABL800 (Radiometer Medical, Tokyo, Japan), while leukocytes were counted by S.K., N.U. and H.I. using an erythrocytometer and the average of their results was calculated.

### Detection of apoptosis

Apoptosis of tumor cells was detected by the TUNEL method using an ApopTag Plus Peroxidase *In situ* Apoptosis Detection kit (Chemicon International, Temecula, CA, USA). Briefly, after deparaffinization and rehydration, samples were pretreated by incubation with proteinase K (2 μg/ml; Merck, Darmstadt, Germany) for 15 min at 37°C. After endogenous peroxidase was inactivated by incubation with 3% H_2_O_2_ in phosphate-buffered saline (PBS) for 5 min, sections were rinsed with PBS and then incubated with terminal deoxynucleotidyl transferase (TdT) buffer containing 1 mM of cobalt-HCl, 0.5 U/l terminal transferase and 0.4 μM of digoxigenin-11-deoxyuridine triphosphate (dUTP) in a humidified chamber for 60 min at 37°C. The reaction was stopped by adding TdT stop buffer, anti-digoxigenin peroxidase conjugate was added, and incubation was carried out for 30 min. As a negative control, slides were incubated without TdT. After visualization of the reaction products with diaminobenzidine (Sigma Chemical Co., St. Louis, MO, USA), nuclei were counterstained with methyl green. Since many tumors showed central necrosis (Fig. 5A-a), even in the control group, we counted the number of apoptotic cells (TUNEL-positive cells) outside the central necrotic area.

### Statistical analysis

Data were analyzed by the Chi-square test and the Mann-Whitney U test using StatFlex version 6.0 software (Artech Co., Ltd., Osaka, Japan). P<0.05 was considered to indicate statistically significant differences.

## Results

**Figure 2:**
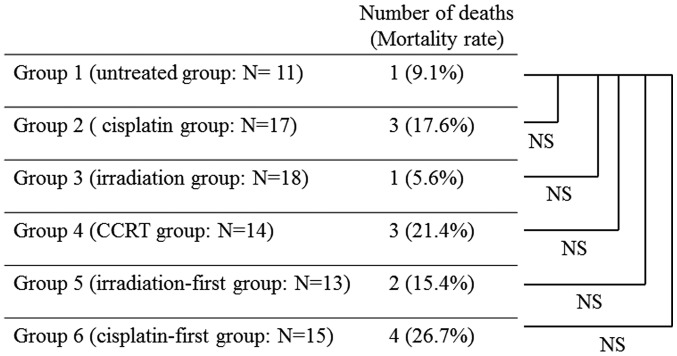
Comparison of the mortality rate between each group. The mortality rate was the highest in Group 6, but there was no significant difference from any of the other groups. NS, not significant.

The number of mice that died before the scheduled day for harvesting specimens was 1/11 (9.1%) in Group 1, 3/17 (17.6%) in Group 2, 1/18 (5.6%) in Group 3, 3/14 (21.4%) in Group 4, 2/13 (15.4%) in Group 5 and 4/15 (26.7%) in Group 6. The mortality rate was the highest in Group 6, but there was no significant difference from the rate in Group 1 (P=0.261; Fig. 2). Mice that died early were excluded from the analysis of the antitumor activity and adverse effects, except mortality. Thus, the number of animals analyzed in each group was 10 in Group 1, 14 in Group 2, 17 in Group 3, 11 in Group 4, 11 in Group 5 and 11 in Group 6.

**Figure 3:**
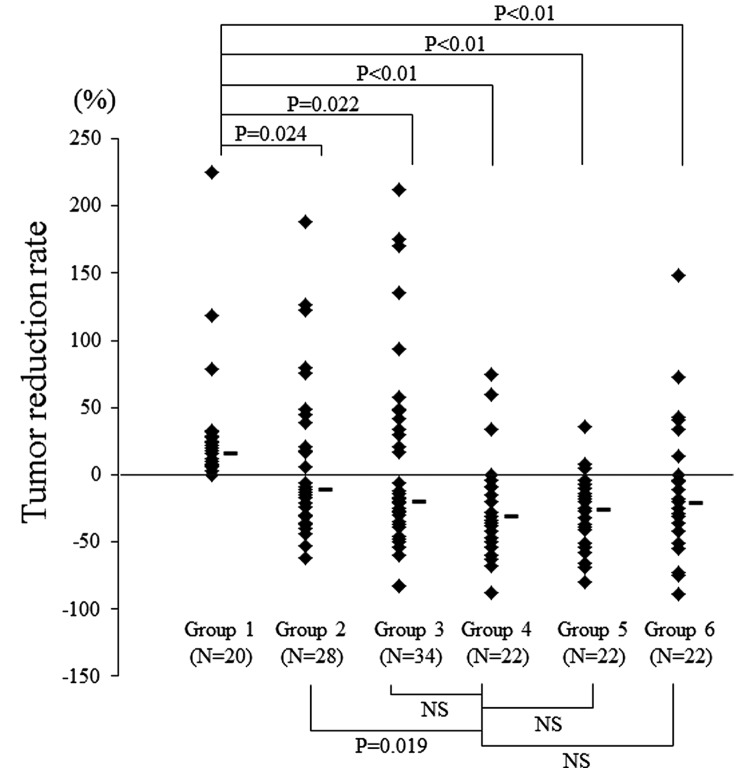
Tumor reduction rate. In Group 4, tumors showed the greatest reduction in size. There was a significant difference between Group 4 and Group 2 (P=0.019), but not between Group 4 and Groups 3, 5 or 6. NS, not significant.

**Figure 4:**
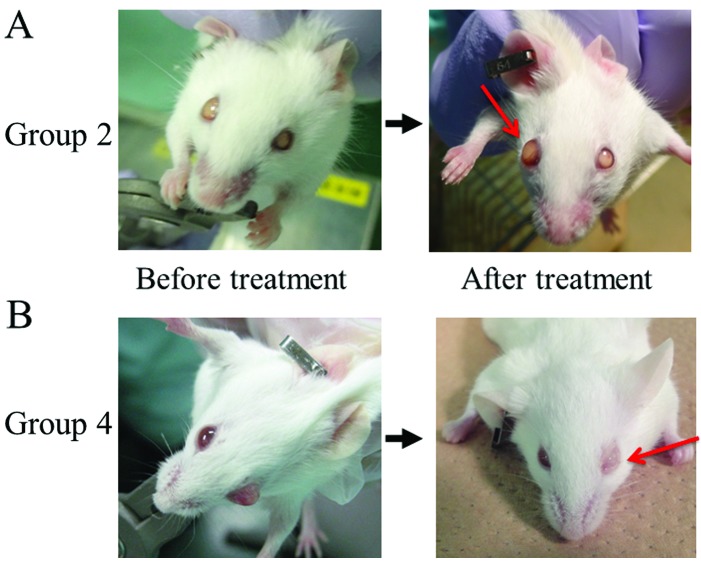
Representative images of mice from Groups 2 and 4 before and after treatment. (A) A mouse from Group 2. The eyeball is larger after treatment than before treatment. (B) A mouse from Group 4. The eyeball showed a marked decrease in size after treatment.

**Figure 5:**
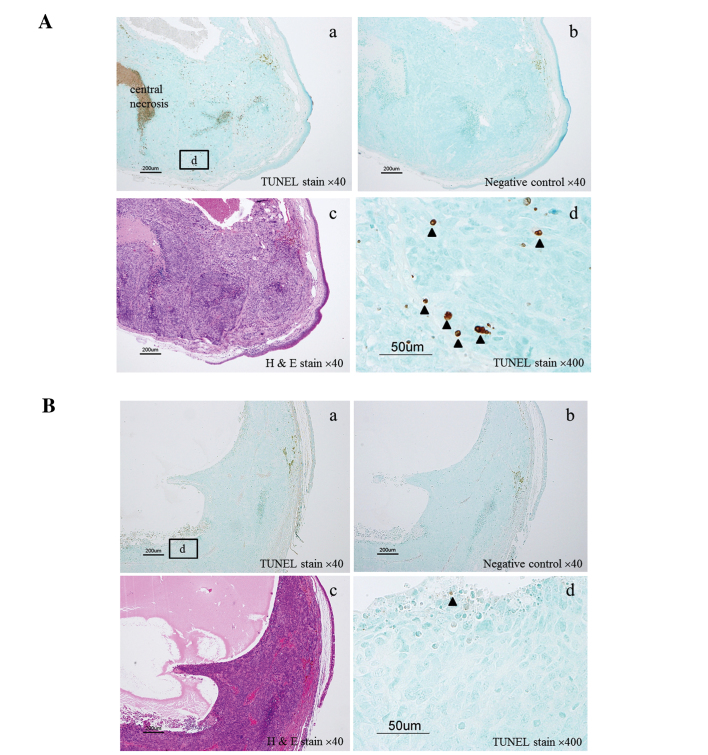
Detection of TUNEL-positive cells in lens tissue. (A) Eyeball of a mouse from Group 4 with numerous TUNEL-positive cells in the lens tissue. (B) Eyeball of a mouse from Group 2 with few TUNEL-positive cells in the lens tissue. a, TUNEL staining, magnification, ×40; b, negative control, magnification, ×40; c, H&E staining, magnification, ×40 and d, TUNEL staining, magnification, ×400.

**Figure 6:**
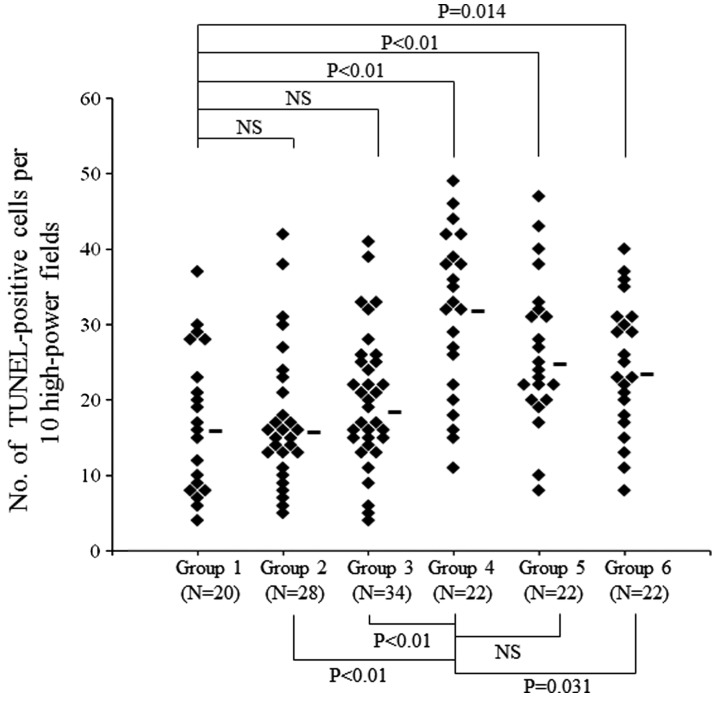
The number of TUNEL-positive cells per 10 high-power fields in lens tissue. The number of TUNEL-positive cells was the highest in Group 4, and there was a significant difference between Group 4 and Groups 2, 3 or 6 (P<0.01, P<0.01 and P=0.031, respectively). NS, not significant.

In each group, antitumor activity was assessed by comparison of the tumor diameter reduction rate between before and after treatment (Fig. 3). In Group 4, the tumors showed the most marked decrease in size and there was a significant difference between Group 4 and 2 (P=0.019), although there was no significant difference between Group 4 and Groups 3, 5 or 6. Representative images obtained from Groups 2 and 4 before and after therapy are shown in Fig. 4. We also evaluated apoptosis in each group to examine the effect of treatment. Apoptotic cells were defined as TUNEL-positive cells with obvious nuclear immunoreactivity (Fig. 5A and B). Immunohistochemical analysis revealed that the median number of TUNEL-positive cells in lens tissue per 10 high-power fields was 15.5 (range, 4–37) in Group 1, 15.5 (range, 5–42) in Group 2, 18 (range, 4–41) in Group 3, 32 (range, 11–49) in Group 4, 24.5 (range, 8–47) in Group 5 and 23 (range, 8–40) in Group 6. The number of TUNEL-positive cells was the highest in Group 4, and there was a significant difference between the number in Group 4 and that in Groups 2, 3 or 6 (P<0.01, P<0.01 and P=0.031, respectively) (Fig. 6).

**Figure 7:**
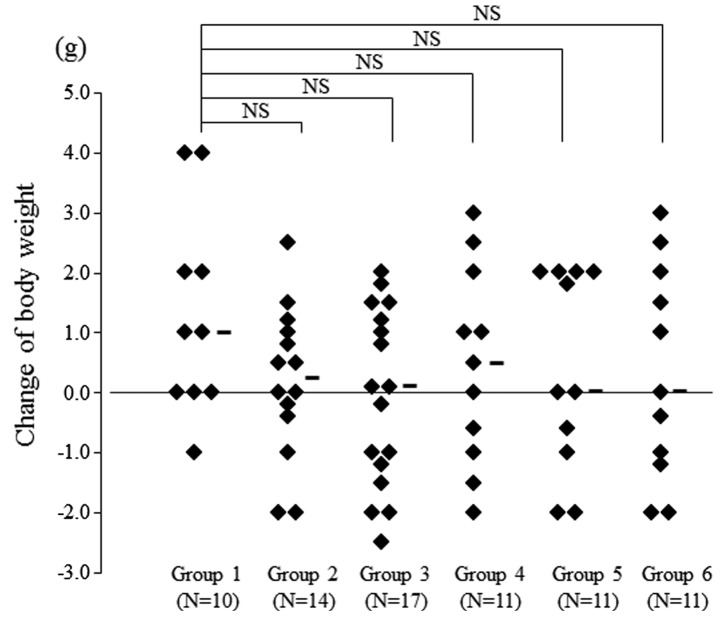
Changes of body weight. Comparison of the changes of body weight showed that there was no significant difference between Group 1 and any of the other groups. NS, not significant.

**Figure 8:**
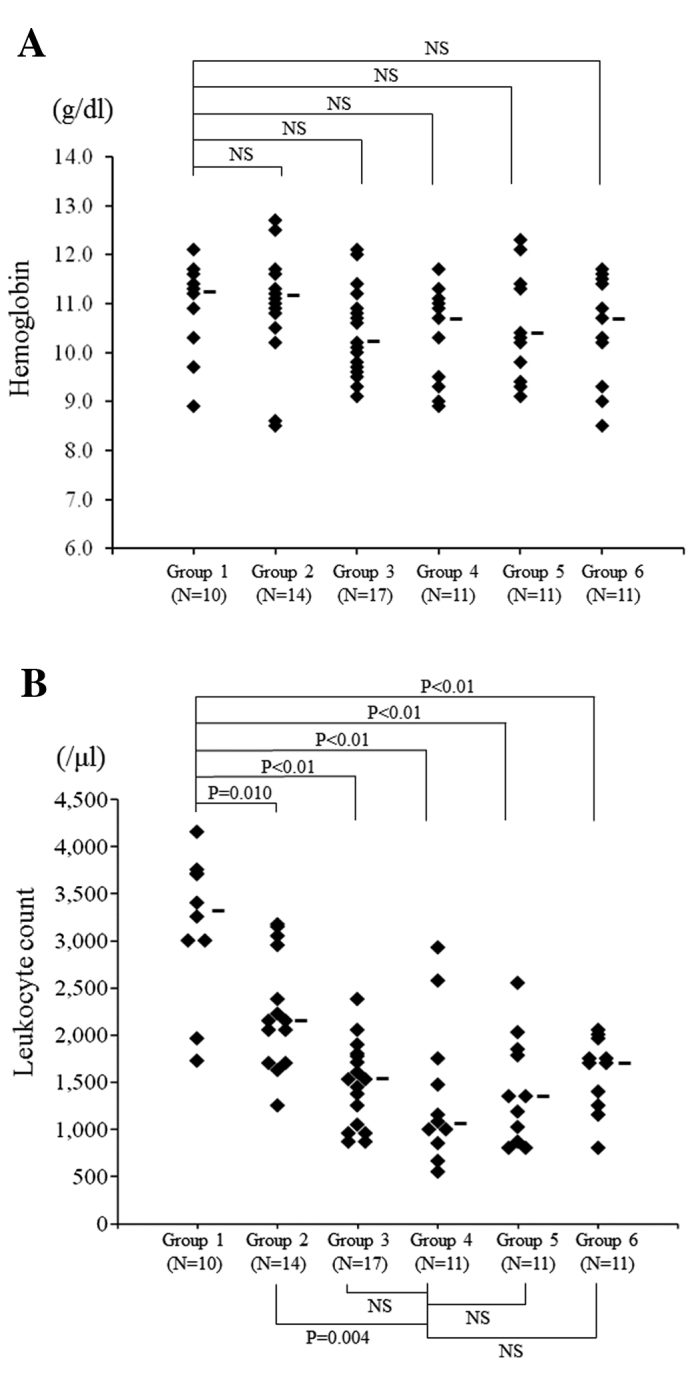
Hemoglobin and leukocyte count. (A) Comparison of the hemoglobin. There was no significant difference of hemoglobin between Group 1 and any of the other groups. (B) Comparison of the leukocyte count. Group 4 had the lowest leukocyte count and there was a significant difference compared with Group 2 (P=0.004), but there was no significant difference between Group 4 and Groups 3, 5 or 6. NS, not significant.

Finally, we compared the adverse effects of each regimen. Investigation of the changes of body weight showed that there were no significant differences between Group 1 and any other group (Fig. 7). Therefore, the anorectic effect of treatment did not show marked differences among the groups. In addition, we examined the severity of myelosuppression by measuring the hemoglobin and leukocyte count after treatment. We found that the hemoglobin did not show a significant difference between Group 1 and any of the other groups (Fig. 8A). The leukocyte count was the lowest in Group 4 and there was a significant difference compared with Group 2 (P=0.004), but there was no significant difference between Group 4 and Groups 3, 5 or 6 (Fig. 8B).

## Discussion

To improve the outcome of treatment for locally advanced cervical cancer, radiotherapy has become mainstream and, to increase the effect of radiation chemotherapy, agents are administered before radiotherapy, concurrently with radiotherapy or after radiotherapy. However, only concurrent chemoradiation has been proven to improve disease-free survival and overall survival in patients with cervical cancer (8–11), while there have been a number of reports that performing chemotherapy before radiotherapy does not improve survival (22–26). The anticancer drug that has proved to be most effective with radiation is cisplatin either alone or in combination with other agents such as 5-fluorouracil (4–7). Therefore, CCRT with cisplatin has become the standard treatment for locally advanced cervical cancer.

The effect of irradiation is presumably enhanced by performing concurrent chemotherapy due to a radiosensitizing effect of anticancer drugs that enhances initial radiation damage to DNA. For example, cisplatin interacts with nucleophilic sites on DNA or RNA to form intra-and inter-strand crosslinks (12). Second, chemotherapy agents inhibit cellular repair processes and exacerbate radiation damage. Grégoire *et al* reported that the effect of fludarabine on radiocurability in mice was greater when it was combined with fractionated radiation than when it was combined with a single dose of radiation (27). Third, chemotherapy can cause the accumulation of cells in the radiosensitive phases of the cell cycle (the G2 and M phases) or eliminate cells in the radioresistant phase (S-phase) (28–30). Some *in vitro* studies using human cervical squamous cell carcinoma cell lines have already investigated the timing of anticancer drug administration (15,16). Tanaka *et al* reported that sensitivity to nedaplatin was enhanced by irradiation and this effect was significantly greater when cells were treated 8 h before or 8 h after irradiation than when they were treated concurrently with irradiation (16). They also reported that 5 of the 6 etoposide-resistant subclones established from ME180 cells showed significant radioresistance, indicating that etoposide should be administered to patients with advanced cervical squamous cancer after the completion of radiotherapy (15).

Although it is inevitable that CCRT will be associated with enhanced acute toxicity (12,13), there has been a lack of animal studies on the relation between adverse effects and the timing of administration of anticancer drugs. Therefore, we performed the present investigation using αT3 transgenic mice bearing SV40-induced undifferentiated lens epithelial tumors (17,18). Comparison of the three combined treatment groups showed that the antitumor activity of CCRT was superior with respect to the tumor reduction rate and the apoptotic effect, although leukopenia was also most severe. In contrast, when cisplatin was administered before radiotherapy the antitumor activity (both tumor reduction rate and the apoptotic effect) was lower than with CCRT or with administration of cisplatin after radiotherapy, and there was a significant difference in the extent of apoptosis between the CCRT group and the cisplatin-first group (P=0.031). Although leukopenia was less severe in the cisplatin-first group, there was no significant difference from the other groups. These results suggest that it may be unwise to administer cisplatin before radiotherapy. It was recently reported that neoadjuvant chemotherapy with weekly paclitaxel and carboplatin before CCRT is beneficial for locally advanced cervical carcinoma (31,32). Therefore, further studies are needed to examine the effectiveness of such agents with radiotherapy in our animal model.

In conclusion, the present study performed on mice did not show the superiority of CCRT over administration of chemotherapy after radiotherapy with respect to efficacy and adverse effects, therefore we could not demonstrate that CCRT is the optimum treatment. However, our findings in this animal model demonstrated that chemotherapy with cisplatin should probably not be performed before irradiation for the treatment of cancer.
